# Efficacy and Safety in the Implantation of Totally Implantable Venous Access Devices by Anesthesiologists: Perspectives From a Retrospective Oncology Cohort

**DOI:** 10.7759/cureus.74606

**Published:** 2024-11-27

**Authors:** Nicolás Valls, Nicolás Villablanca, Roberto González, María Soledad Ramirez, Carla Almeida, Julio Lopez

**Affiliations:** 1 Anesthesiology, Anesthesia Unit, National Cancer Institute, Santiago, CHL; 2 Anesthesiology and Perioperative Medicine, University of Chile Clinical Hospital, Santiago, CHL; 3 Anesthesiology, Anesthesia Unit, Coyhaique Regional Hospital, Coyhaique, CHL

**Keywords:** anesthesiologist, chemotherapy, clinical oncology, perioperative complications, vascular access

## Abstract

Background: Totally implantable venous access devices (TIVADs) are widely used in oncology patients to facilitate central venous access. Although they offer benefits, TIVADs can be associated with complications.

Materials and methods: This retrospective cohort study included all oncology patients 18 years or older who underwent TIVAD implantation between September 2015 and October 2019. Data were obtained from clinical records at the National Cancer Institute.

Results: A total of 556 TIVAD implantations were performed in cancer patients throughout the study period. The success rate for the first attempts was 91% (506/556). Infectious complications were documented in six patients (1.1%), while non-infectious complications manifested in less than 1% of cases, with hematoma at the insertion site being the most common. Additionally, catheter thrombosis was identified in three asymptomatic patients (0.5%).

Conclusion: The implantation of TIVADs by anesthesiologists in cancer patients at the National Cancer Institute was predominantly successful and safe, exhibiting a low complication rate. The findings reinforce the efficacy and safety of the employed technique, exceeding the outcomes reported in existing medical literature.

## Introduction

Totally implantable venous access devices (TIVADs), also commonly referred to as Port-a-Cath®, have been a cornerstone in clinical practice for an extended period. Since their inception in 1982, the utilization of these devices has experienced exponential growth. TIVADs, comprising a reservoir housed in subcutaneous tissue and a catheter that connects to a central vein, are crucial for patients with limited peripheral venous access who require long-term continuous administration of medications. Their primary benefit is that they provide intermittent and repetitive central venous access, allowing for ambulatory administration of antineoplastic drugs, blood sample collection, intravenous medication delivery, and even parenteral nutrition administration. The advent of TIVADs has revolutionized cancer patient care by offering long-term, safe, and efficient central venous access. However, despite extensive experience and a plethora of literature investigating factors associated with TIVAD outcomes, complications during perioperative and postoperative stages remain a constant concern. Indeed, although TIVADs are recognized as the safest form of long-term central venous access, reported general complication rates for these devices range between 2% and 18% of all implanted units [1-5].

Early complications frequently arise due to injuries to adjacent structures during venous puncture, catheter insertion, and reservoir implantation, whereas late complications are typically the result of prolonged catheter dwell time and improper use. Port-related infections and venous thrombosis are particularly concerning as they entail additional morbidity, elevate costs, and often necessitate TIVAD removal, which occurs in up to 6.5% of patients [6].

Although the implantation of TIVADs is a minor surgical procedure usually performed by surgeons, the anesthesiology team at the National Cancer Institute has assumed responsibility for their insertion since 2015. This medical-surgical procedure can entail complications, so understanding the most common ones is essential. In this article, we will delineate our five-year experience concerning complications arising from the implantation technique and the continual use of TIVADs in patients at the National Cancer Institute.

## Materials and methods

Before conducting the study, approval was obtained from the Scientific Ethics Committee of the Metropolitan Eastern Health Service (Approval No.: 035/219). A retrospective cohort study was designed to describe the technique and rate of complications associated with the installation of TIVADs. All patients aged over 18 years who had a TIVAD installed at the National Cancer Institute between September 2015 and October 2019 were included in the study. Data were extracted from the clinical records registry.

Regarding statistical analysis, the sample size results from the consecutive inclusion of patients during the specified period. For descriptive statistics, continuous variables with normal distribution were expressed as the mean and standard deviation, while categorical variables were expressed as absolute frequency and percentage. Tables and statistical analyses were performed using RStudio (Boston, MA) and GraphPad Prism v8.0 (La Jolla, CA).

Technique description

The implantation of TIVADs is coordinated through the institution's preoperative unit, where the patient's medical history is reviewed, focusing on anticoagulants or antiplatelet agents, and any necessary treatment interruptions or adjustments are made. In addition, general laboratory tests such as platelet count and coagulation tests are consulted.

Initially, informed consent is obtained from each patient. Subsequently, the implantation procedure is performed with the patient in the supine position, slightly in Trendelenburg, and under non-invasive monitoring (electrocardiogram, non-invasive blood pressure, and oximetry). Rigorous aseptic technique and local anesthesia are employed. Venous puncture of the selected vein is conducted under ultrasound guidance, and a metallic guide wire is subsequently introduced using the Seldinger technique, confirming proper direction through electrocardiographic changes. A subcutaneous pocket, usually in the infraclavicular region over the pectoral muscle, is formed to house the device's reservoir.

In specific cases, fluoroscopy was employed to enhance precision, particularly during the advancement of the guidewire and the placement of the catheter within the vascular system. When fluoroscopy was utilized, our approach aligned with the technique described by Granziera et al. [4]. We aim to confirm the catheter's position at the cavoatrial junction, considering the catheter tip to be optimally placed when it is located within 2 cm of the lower border of the right main bronchus. Next, subcutaneous tunneling connects the reservoir to the catheter positioned within the vascular system. The determination of catheter length was primarily guided by the clinical experience of the medical team, who considered factors such as patient height, gender, and puncture site. The catheter length was measured using the markings on the catheter itself. This approach draws on a literature review and employs a technique similar to the topographical method described by Ezri et al. [7]. This method involves an external measurement along the body's surface, extending from the insertion point to the manubriosternal joint or the second to third intercostal space level. Then, the correct blood flow and reflux from and into the chamber were verified, and finally, the device was irrigated with a sodium heparin solution. To conclude the procedure, a chest radiograph is performed to verify the correct position of the catheter's distal end and detect potential complications arising from the intervention.

Sedation or general anesthesia is reserved for patients who exhibit significant anxiety or are unable to tolerate the procedure under local anesthesia. In these instances, a team consisting of an anesthesiologist and a specialized operator administered the sedation or anesthesia.

## Results

Between September 2015 and October 2019, 556 TIVADs were implanted by the anesthesiology team. Of these, 324 (58%) were female patients with an average age of 53 ± 15 years. The most frequent diagnosis necessitating the TIVAD implantation was breast cancer, accounting for 165 cases (29.7%). Secondary frequent oncological diagnoses were colon cancer and gastric cancer, comprising 75 (13.5%) and 61 (11%) patients, respectively. Regarding the type of anesthesia used, local anesthesia with 1% lidocaine was employed in 546 patients, representing 98% of the cases. General anesthesia was required to complete the procedure in only two patients (0.4%), and the procedure was carried out under sedation in four patients (0.7%), as shown in Table 1.

**Table 1:** Demographic and clinical characteristics of patients undergoing TIVAD insertion (N = 556) The table accounts for patient demographics, including age and gender, as well as specific diagnostic categories. It outlines the types of anesthesia used, the anatomical sites chosen for venous access, the number of attempts made for successful insertion, and the utilization of radiographic guidance. Data are summarized as mean and standard deviation (SD) for age and as absolute numbers (N) and percentages (%) for all categorical variables.

Variables	
Age mean ± SD	53 ± 15
Female sex, N (%)	324 (58)
*Diagnosis*	
Breast cancer, N (%)	165 (29.7)
Colon cancer, N (%)	75 (13.5)
Gastric cancer, N (%)	61 (11)
Non-Hodgkin lymphoma, N (%)	52 (9.4)
Rectal cancer, N (%)	48 (8.6)
*Type of anesthesia*	
Local, N (%)	550 (98.9)
General, N (%)	2 (0.4)
Sedation, N (%)	4 (0.7)
*Access site*	
Right internal jugular, N (%)	285 (51.3)
Left internal jugular, N (%)	127 (22.8)
Left subclavian, N (%)	70 (12.6)
Right subclavian, N (%)	34 (6.1)
Right axillary, N (%)	29 (5.2)
Left axillary, N (%)	9 (1.6)
Femoral, N (%)	2 (0.4)
*Number of attempts*	
First attempt, N (%)	506 (91)
Two attempts, N (%)	26 (4.7)
Without fixation, N (%)	529 (95.1)
Without fluoroscopy, N (%)	502 (90.3)

The most frequently punctured site for venous access was the right internal jugular vein, accounting for 285 cases (51.3%), followed by the left internal jugular vein in 127 patients (22.8%). The procedure was successful on the first attempt in 506 cases (90.5%). On the other hand, intraprocedural fluoroscopy was necessary to evaluate the advancement of the guide wire in 54 patients (9.7%). The TIVAD implantation technique was considered successful in 545 cases (98%); in the remaining 11 patients (2%), catheter repositioning was necessary due to incorrect placement of the distal end, as confirmed by chest radiography. If the catheter was longer than necessary, it was withdrawn under sterile conditions, and the distal tip's position was corrected with fluoroscopic guidance. if the catheter was too short, resulting in a more proximal-than-optimal placement, the procedure was restarted entirely. This involved removing the original catheter and inserting a new one, with fluoroscopy used to guide the new placement attempt.

Regarding non-infectious complications, the most frequent was hematoma at the puncture site, occurring in nine patients (1.6%). The remainder of the complications appeared in less than 1% of the patients (Table 2). Notably, pneumothorax occurred only in two patients (0.4%). No other complications are commonly described in the literature, such as air embolism, hemorrhagic events due to laceration of large vessels, arrhythmias, or injuries to the esophagus, trachea, lymphatics, and brachial plexus. On the other hand, during the follow-up, it was observed that two patients (0.4%) exhibited dehiscence of the suture in the subcutaneous pocket, necessitating the removal of the catheter and its reservoir.

**Table 2:** Complications associated with the insertion of TIVADs The table provides an overview of the frequency and types of complications experienced by patients who underwent TIVAD insertion based on a total sample size of 556 cases. Data are presented as absolute numbers (N) and percentages (%).

Complication category	
No complications, N (%)	515 (92.8)
Any complication, N (%)	41 (7.2)
Type of complication	
Hematoma, N (%)	9 (1.6)
Late infection (>30 days), N (%)	4 (0.7)
Thrombosis, N (%)	3 (0.5)
Exteriorization, N (%)	3 (0.5)
Pneumothorax, N (%)	2 (0.4)
Early infection (<30 days), N (%)	2 (0.4)
Suture dehiscence, N (%)	2 (0.4)
Extravasation, N (%)	2 (0.4)
Reservoir rotation, N (%)	1 (0.2)
Obstruction, N (%)	1 (0.2)
Others, N (%)	11 (2.0)

As for other non-infectious complications, the most frequent was the migration of TIVAD, observed in four cases, which corresponds to 0.7% of the patients. Three manifested as the reservoir's externalization and one as the reservoir's rotation, also known as Twiddler's syndrome. Catheter thrombosis was observed in three patients (0.5%), with none exhibiting symptoms or an associated clinical picture. All were diagnosed through computed tomography and appeared as findings in the follow-up of the underlying oncological disease. On the other hand, there were reports of drug extravasation in two patients (0.4%), which, upon further case evaluation, were determined to be due to poor needle positioning during TIVAD manipulation for infusion and catheter fissure associated with over-pressurization of the reservoir.

In terms of infectious complications, these were observed in six patients, accounting for 1.1% of the total sample. Of these, in two patients (0.4%), the infection manifested within the first 30 days following the TIVAD installation, whereas in four patients (0.7%), the infection occurred after 30 days had elapsed.

## Discussion

In the present study, we report the outcomes from September 2015 to October 2019, during which 556 TIVAD installations were carried out on oncologic patients. Overall, the procedure was both successful and safe. Although complications were recorded, their incidence was low. A success rate of 91% (506/556 patients) was achieved on the first attempt. Infectious complications were seen in six patients (1.1%). Furthermore, non-infectious complications were observed in less than 1% of the cases. Therefore, our results highlight the effectiveness and safety of the procedure.

Infection is one of the most common and certainly among the most severe complications associated with the use of TIVADs. It is linked to increased morbidity and mortality rates, treatment delays, extended hospitalization, and elevated healthcare costs. Our findings are consistent with existing literature, but they underscore a relatively low rate of infectious complications. While the result is promising, it remains crucial to maintain rigorous aseptic protocols during both implantation and usage phases to minimize this critical risk factor [8]. Preventive measures are of vital importance in mitigating the risks associated with TIVADs. Clinical guidelines strongly support and recommend using specific protocols for infection prevention during both the insertion and maintenance phases. An ideal insertion protocol should include a dedicated environment for the procedure, adequate hand hygiene, skin antisepsis, barrier precautions, ultrasound-guided venous puncture, appropriate site selection for the reservoir, and proper skin closure. An ideal maintenance protocol should encompass hand hygiene, sterile gloves, skin antisepsis before inserting the Huber needle, aseptic handling of the infusion line, and needle replacement within one week. Despite the implementation of standardized protocols and concerted efforts, surgical site infection remains the most frequent complication in patients undergoing surgery [8,9], a scenario reflected in our analysis. It is critical to emphasize that, in our case, the intervention involves the implantation of devices that contain an intravascular segment and are frequently manipulated for clinical use, increasing the likelihood of infection. In our records, only two infections occurred within the first 30 days, which could be associated with the implantation procedure.

Consequently, a postoperative follow-up was initiated with the nursing team during the first week following surgery. The team continued to monitor cases that required it and referred them for medical treatment if complications were identified. From its implantation, the chemotherapy unit prepares the catheter for use. As such, improper handling cannot be ruled out as a cause of early infections. Most infections occurred after the 30-day mark, suggesting a direct correlation with the manipulation and usage of the TIVAD.

This finding spurred a collaborative effort with other units that regularly use the device to establish minimum standards in TIVAD handling to reduce perioperative infection cases. Notably, infectious complications affected only six patients (1.1%), significantly lower than the 4.8% reported in the literature [10].

Among the strategies that have improved safety in the clinical practice of implantable catheter insertion is the widespread use of ultrasonography during the various phases of TIVAD insertion. Ultrasound-guided venipuncture is considered mandatory for any central venous catheterization. This technique allows for preliminary evaluation of the veins, real-time venipuncture, and early detection of complications such as hematoma and pneumothorax [11]. Furthermore, ultrasonography is part of the SIC (Safe Insertion of Centrally Inserted Central Catheters) protocol described by Brescia et al. [12], which recommends evaluating and selecting the appropriate central vein and optimal exit site location in the cervicothoracic region and their possible combinations to ensure a lower risk of infection. Another relevant complication concerning TIVADs is obstruction and catheter-associated thrombosis.Our findings showed a low incidence of venous thrombosis related to TIVADs, affecting only three patients (0.5%). This low rate could be linked to the choice of access route, as suggested by other authors. Evidence indicates that the incidence of catheter-related thrombosis can vary depending on the catheter tip's anatomical location and the insertion technique. Therefore, the low incidence of thrombotic complications in our cohort may well underscore the effectiveness of our procedural protocols [12].It is worth noting that none of these patients displayed symptoms or presented with clinical manifestations related to thrombosis. Detection was incidental through computed tomography scans during the follow-up of their underlying oncological disease. This finding is consistent with current literature, which reports that most cases of venous thrombosis related to TIVADs are asymptomatic. Despite this, it is crucial to underline the importance of detecting these thromboses due to the potential associated complications, such as pulmonary embolism and an increased risk of catheter-related infections.

It is essential to emphasize that catheter obstruction is a frequent issue, often caused by mechanical factors such as pinch-off syndrome, drug accumulation, incompatible drug combinations, or clots within the catheter. Catheter obstruction detection usually occurs when it becomes impossible to draw blood using negative pressure (withdrawal occlusion) or when difficulties arise in infusing with positive pressure (complete occlusion). The diagnostic approach will depend on the underlying cause. Inspection and repositioning maneuvers may be attempted to resolve temporary mechanical obstructions. Moreover, it is crucial to examine the compatibility of administered drugs meticulously. Once these factors are ruled out, thrombosis becomes the most common cause of obstruction. Regrettably, managing venous thrombosis related to TIVADs remains a topic of debate due to the need for more prospective studies. Current American College of Chest Physicians guidelines recommend acute management with low-molecular-weight heparin for three months or continuously while the TIVAD remains in place. The guidelines also suggest that TIVADs should only be removed if they are no longer needed or are not functioning correctly [13,14].

The classical technique for the implantation of TIVADs is guided by fluoroscopy, which allows the physician to visualize the catheter's path in real-time, ensuring its proper position within the vein [14]. Fluoroscopy is an acceptable intraoperative method, but it is often imprecise, expensive, logistically challenging, and even unsafe, as it exposes patients and operators to ionizing radiation. Intracavitary ECG is the most cost-effective and precise intraoperative diagnostic method for tip localization [15]. Another effective, economical, and non-invasive intraprocedural method for tip localization is transthoracic echocardiography using the "bubble test" (a rapid infusion of a few milliliters of "agitated" saline solution that allows for better visualization of the catheter tip) [16]. However, within our group, these methods are not commonly practiced; instead, we monitor electrocardiographic changes in the advancement of the guidewire. This approach did not result in adverse events associated with physical stimulation of the right atrium, particularly arrhythmias.

Similarly, the incidence of mispositioning of the catheter tip was low, and reinstallation of the device was required only in isolated cases. In this regard, and as indicated by our results, the participation of the imaging unit is optional for carrying out the procedure except in specific cases. This reduces the human resources, infrastructure, and time required to install TIVADs.

Finally, most complications can be prevented, as they usually arise due to errors during the device implantation or inadequate postoperative care [3]. For this reason, it is crucial to establish standardized protocols for the installation procedure and develop systems for detecting and intervening complications as they arise. In our center, improvement measures were implemented once cases with complications were detected. For example, the venous access site was changed from subclavian to jugular to avoid pneumothoraxes, which had previously occurred with subclavian access. Additionally, a change was made in the closure technique, transitioning from a simple interrupted suture to a Donati stitch, which reduced the cases of implant extrusion and loss. Likewise, with the acquisition of experience, there was a decrease in the incidence of hematomas and arterial punctures.

The main limitation of our study is that it corresponds to an analysis with a short follow-up period for our patients, with a maximum of five years, from the installation of the first TIVAD by the anesthesiology team to the conduct of this study. Therefore, many late complications may still need to be detected if the follow-up period is extended.

Anesthesiologists are trained to insert central venous access, which demands technical skill and the competency to identify and manage associated complications. However, the implantation of TIVADs has traditionally been a function mainly carried out by surgeons. Despite this, other medical teams can assume this responsibility with appropriate training. At the National Cancer Institute, the Department of Anesthesiology decided to take on this challenge. The initiative began with a training period and the support of surgical teams until the necessary skills and experience were acquired to perform the procedure independently. As a result of this initiative, the capability to implant TIVADs doubled compared to the historical records of our hospital.

## Conclusions

The installation of TIVADs by the anesthesia team in oncology patients at the National Cancer Institute was safe and successful in most cases, with a low incidence of complications. The results support the efficacy and safety of the technique used. Our success rate for installing and monitoring TIVADs is comparable to that of medical-surgical specialties, and, in fact, our complication rate is lower than that reported in the existing literature.
